# Establishment of a biomarker of peripheral stress in opioid addicts based on the hypothalamic-pituitary-adrenal axis—The improvement effect of exercise

**DOI:** 10.3389/fpsyt.2022.1072896

**Published:** 2022-12-07

**Authors:** Jingsong Wang, Zhibing Zou

**Affiliations:** Xiangsihu College, Guangxi University for Nationalities, Nanning, China

**Keywords:** stress, craving, exercise, OT, AVP, cortisol

## Abstract

**Purpose:**

This study aims to investigate the relationship between peripheral blood oxytocin (OT), vasopressin (AVP), and subjectively perceived stress and cortisol in male opioid addicts based on the hypothalamic-pituitary-adrenal (HPA) axis. We also investigate the impact of exercise on reducing subjectively perceived stress, craving level, negative reinforcement, anxiety, sleep quality, plasma OT, AVP, and cortisol levels.

**Methods:**

Participants were divided into 28 subjects in the low-stress control group (LSC group), 29 subjects in the medium-high stress control group (MTHSC group), and 28 subjects in the moderate-high-stress exercise group (MTHSE group), based on their subjectively perceived stress levels. Subjects in the MTHSE group performed 12 weeks of combined aerobic resistance training (60 min per day, 5 days per week). Plasma OT, AVP, and cortisol concentrations were analyzed *via* Elisa. PSQI was used to assess the subjective perceived stress, craving, negative reinforcement, anxiety, and sleep quality level, respectively. Mixed-effects ANOVA and Pearson correlation analysis were employed to explore the impact and correlation between different parameters.

**Result:**

Plasma OT levels significantly increased (95% CI: –7.48, –2.26), while plasma AVP (95% CI: 2.90, 4.10), and cortisol (95% CI: 19.76, 28.17) levels significantly decreased in the MTHSE group after exercise. The PSS (95% CI: 1.756, 4.815), “Desire and Intention” (95% CI: 1.60, 2.71), and “Negative reinforcement” (95% CI: 0.85, 1.90) (DDQ), SAS (95% CI: 17.51, 26.06), and PSQI (95% CI: 1.18, 3.25) scores of the MTHSE group were significantly decreased after exercise. Plasma OT, plasma cortisol, craving, negative reinforcement and anxiety were negatively correlated. Plasma AVP was positively correlated with craving.

**Conclusion:**

As an auxiliary treatment, exercise improves the plasma OT, AVP, and cortisol levels of opioid addicts, and reduces their subjective perceived stress level, desire, negative reinforcement level, anxiety level, and sleep quality. In addition, peripheral plasma OT, AVP, and cortisol may play a role as potential peripheral biomarkers to predict stress in male opioid addicts.

## Introduction

Opioid use disorder is the compulsive usage of various opioids substances and is a general term for various addictive mental disorders resulting from opioids substances. Its etiology is more complex and results from the interaction of psychological, social and biological factors. It is currently generally accepted that, along with genetic factors, the external stressful environment of human life is also a risk factor for opioid use disorder (1). It may clearly be of importance to decrease the stress levels of opioid use disorder patients using predefined treatment methods to predict the change in their degree of addiction.

Stress is a vital factor in causing relapse (2), and stress exposure is associated with the severity of substance use disorder (3). When individuals encounter pressure in their daily lives, it will not only affect their quality of sleep (4) but will also lead to strong negative emotions such as anxiety (5) and the use of drugs to cope with it. Several researchers have put forward two independent views. One is that stressors lead to drug use, For example, both acute and chronic stress exposure have been demonstrated to disrupt PFC network connections and markedly impair PFC functions that are critical for self-control has suggested that uncontrollable stressors may produce particularly powerful deficits (6). And the other is that the self-stress balance changes during chronic drug use. For example, the stress neural adaptation model believes that chronic drug use will lead to continuous changes in pressure distribution to maintain body balance in the case of frequent drug use. This may be due to the slow response of HPA axis pressure source and the sensitivity of CRH nerve transmission in the extended amygdala after long-term drug administration (7). The exploration of a stress-relapse mechanism is of great importance in the field of drug abuse, but it is undeniable that if stress is improved, the severity of drug use can be alleviated.

Increasing evidence that alterations and dysregulation of the hypothalamic-pituitary-adrenal (HPA) axis and other neuropeptides can potentially predict addiction relapse (8). Psychoactive substances (methamphetamine, heroin, etc.) are actively involved in the arousal pathway of stress (9). The physiological response to stress is mediated by the HPA axis, which is in turn mediated by the paraventricular nucleus of the hypothalamus, pituitary gland, and adrenal gland composition. Neurons in the paraventricular nucleus synthesize and release a corticotropin-releasing factor (CRF) into the portal vein of the anterior pituitary. CRF binds to the CRF1 receptor on the pituitary corticotropin, which induces adrenocorticotropic hormone (ACTH) into the peripheral circulation. ACTH then stimulates the adrenal glands to synthesize and secrete glucocorticoids (cortisol), which modulate the body’s stress response (10). ACTH also regulates the release of corticotropin-releasing hormone (CRH), followed by OT and AVP.

Researchers have studied the concentration of corticotropin in plasma/serum, cortisol in plasma/serum and saliva of opioid use disorder patients and found that it can be used as an exemplified indicator of chronic stress exposure (11). Several studies have associated plasma/salivary cortisol changes in stress responses to risk factors for mood and addiction disorders in opioid use disorder patients (11–13). Negative reinforcement, refers to drug-taking in order to relieve the kind of negative emotional states (e.g., anxiety, irritability, and irritability). Behavioral switching driven by negative reinforcement is mediated by the HPA axis and the brain stress system (corticotropin-releasing factor; CRF) in the amygdala (14–16). On the HPA axis, stress increases the release of the corticotropin-releasing factor (CRF) and arginine vasopressin (AVP) from the terminal paraventricular nucleus (PVN) of the hypothalamus into the pituitary portal circulation (17). The above indicators can be utilized as an important example of the stress degree of drug addicts to predict the development of the degree of addiction. The premise is based on the correlation between pressure-related indicators and the degree of addiction.

It is well known that oxytocin and vasopressin have opposite effects in inhibiting and promoting anxiety and stress responses (18–20). Oxytocin may also reduce stress-induced relapse in individuals with heroin addiction (21). Peripheral oxytocin administration has been shown to decrease the elevation of corticosterone (CORT) levels and stress-induced activation of the HPA axis, reducing anxiety and depression levels (18–20). On the other hand, knockdown mice lacking the oxytocin (OTKO) gene exhibited more anxiety-like behaviors and higher CORT responses after exposure to stressful situations (21, 22). AVP is known to potentiate the HPA axis response to stress (23). Under stressful conditions, AVP is co-released with CRF in the hypothalamus potentiating its actions (24). Acute i.c.v administration of AVP causes increased scratching and grooming (behaviors related with stress and anxiety) in wild type but not in V1A receptor-knockout mice (25). Released AVP and OT have opposing effects. AVP promotes a stress response, while OT has a buffering effect in reducing a stress response (26). Intranasal oxytocin has been shown to reduce social stress to some extent (27). Oxytocin can reduce addictive behaviors related to stress and anxiety to a certain extent; it is expected that oxytocin can be applied as a therapeutic target to treat stress-induced addictive behaviors. Although the role of AVP in activating the HPA axis *per se* appears to be limited, AVP can significantly increase the effects of CRF (28–30).

Exercise is widely used as a new type of intervention to improve relapse in drug addicts and is highly valued because it is clean and has no side effects (31). It has been empirically demonstrated that exercise can up-regulate peripheral plasma oxytocin levels and improve social anxiety and cue-induced craving in heroin addicts (32). Although the improvement impact of exercise is relatively optimistic, it cannot be ruled out that some researchers claim that exercise does not have such a great effect (33). Although peripheral oxytocin injection can counteract stress and anxiety in drug addicts and reduce relapse, its peripheral mechanisms remain unknown. Therefore, this study aims to explore the association between peripheral oxytocin and self-perceived stress and craving levels, hypothesizing that exercise can correspondingly improve self-perceived stress and cue-induced craving levels by modulating peripheral oxytocin. The aim is to offer heroin addicts the establishment of peripheral biomarkers for stress perception to provide a theoretical reference for clinical workers and researchers working on the front line.

## Study subjects and methods

### Subjects

This experiment involved 1,260 male compulsory isolation and detoxification workers from the Guangxi Compulsory Isolation Drug Rehabilitation Center, China, as the screening objects. The screening criteria were as follows: (1) All participants satisfied the criteria for heroin dependence according to the structured diagnostic interview of DSM-V; (2) no combined use of other drugs was reported; (3) no mental illness was reported; (4) no bone and muscle disease, cardiovascular and cerebrovascular disease, or immune disease was reported; (5) no history of major trauma and surgery was reported; (6) no alcohol, nicotine, caffeine, beta-blockers, and other substances taken within 6 h before the subjective stress and craving assessment was reported; (7) no regular exercise habits (self-reported moderate-intensity physical activity less than 30 min per day and less than three days per week over three months) were reported, and (8) PAR-Q + (Physical Activity Readiness Questionnaire+) was filled out to ensure that subjects were good for exercise.

**FIGURE 1:**
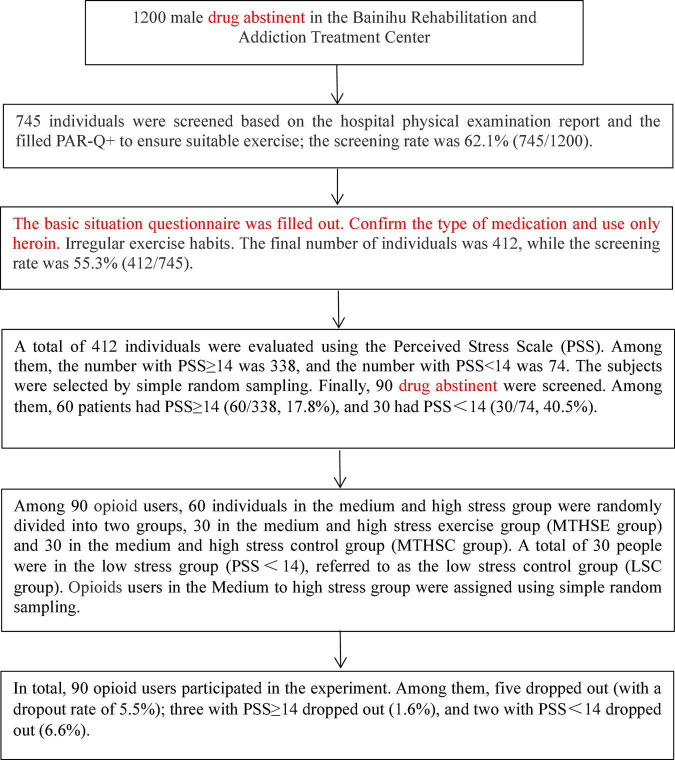
Screening flow chart.

This study complied with the Declaration of Helsinki and was approved by the Ethics Committee of Guangxi Minzu University (Batch number: 16-2010). The rehabilitation center is equipped with fitness equipment with complete facilities, which can meet the exercise needs of the subjects. An informed consent form was filled out for voluntary participation. The reason for the subjects to participate in the experiment was investigated by means of oral interview. The results showed that the subjects had a strong desire to enhance their physical health (internal motivation) and could be appropriately commuted due to their good behavior (external motivation). Among the 90 subjects, 30 were in the low-stress control (LSC) group, 30 were in the medium-high stress control group (MTHSC group), and 30 were in the medium-high stress exercise group (MTHSE group). After excluding those who quit midway, the grouping situation was as follows: 28 individuals in LSC group, 29 in MTHSC group, and 28 in MTHSE group. The dropout rates were 6.6, 3.3, and 6.6%, respectively. There was no difference in PSS scores between MTHSC and the MTHSE groups. See Figure 1 for participant screening process. The basic information about the subjects is shown in Table 1.

**TABLE 1:** Basic information.

Content	LSC group (*n* = 28)	MTHSC group (*n* = 29)	MTHSE group (*n* = 29)
Age (year)	36.14 ± 8.76	36.11 ± 9.36	36.14 ± 9.29
Height (cm)	166.72 ± 5.17	166.89 ± 5.41	167.46 ± 4.34
Weight (kg)	65.79 ± 7.97	63.75 ± 10.42	66.18 ± 7.47
History (month)	73.45 ± 7.79	75.18 ± 12.12	71.11 ± 9.40
The length of drug abstinent (month)	1.63 ± 0.99	1.82 ± 0.99	1.75 ± 1.00
Frequency (week)	9.45 ± 3.86	11.21 ± 6.67	12.14 ± 7.93
Intensity per time (g)	0.40 ± 0.26	0.65 ± 0.52	0.55 ± 0.43
PSS (score)	3.90 ± 2.48	24.54 ± 7.19	25.71 ± 6.81

### Exercise intervention

**FIGURE 2:**
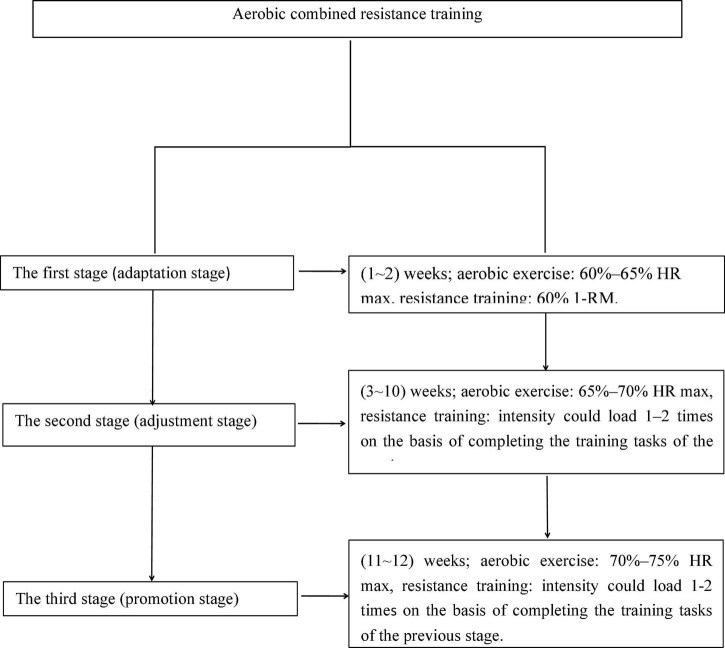
Exercise intervention flow chart.

The subjects were intervened with aerobic combined resistance exercise for 60 min a day, 5 days a week, for a total of 12 weeks. This exercise intervention program follows ACSM’s recommendations (27). The exercise program includes: 5 min warm-up exercise, 30 min treadmill aerobic training, 20 min strength resistance training, 5 min relaxation training. Here, 27 people in the exercise group were subjected to structured and progressive exercise intervention, while the control group received safety and health education. The maximum heart rate (HRmax = 206.9−0.67 × age) and maximum weight (1RM) of each individual were determined before exercise for one repetition. Aerobic exercise was performed on a treadmill Training (Cosmos Pulsar 4.0), The exercises for the muscle groups were divided into the upper limb and lower limb muscle groups, strength resistance training was aimed at improving muscle strength and explosive power, with 8–12 times for each group, two sets for each device, 60 s intervals for each set (Figure 2). A heart rate monitor (Polar V800) was used to continuously monitor the heart rate throughout the training process.

### Exercise intervention

The subjects were intervened with aerobic combined resistance exercise for 60 min a day, 5 days a week, across 12 weeks. This exercise intervention program follows ACSM’s recommendations (34). The exercise program comprises 5 min of warm-up exercises, 30 min of treadmill aerobic training, 20 min of strength resistance training, and 5 min of relaxation training. In total, 27 individuals in the exercise group were subjected to structured and progressive exercise intervention, while the control group received safety and health education. The maximum heart rate (HRmax = 206.9−0.67 × age) and maximum weight (1RM) of each individual were determined before exercise for one repetition. Aerobic exercise was performed on a treadmill (Cosmos Pulsar 4.0). The exercises were divided into the upper limb and lower limb muscle groups. Strength resistance training aimed to improve muscle strength and explosive power, with 8–12 times for each group, two sets for each device, and 60 s intervals for each set (Figure 2). A heart rate monitor (Polar V800) was employed to continuously monitor the heart rate during the training process.

### Procedure

This study was conducted from January to September 2019. In January 2019, we contacted the Drug Rehabilitation and Rehabilitation Center of Guangxi Province. In March 2019, a questionnaire survey about PAR-Q was conducted among male patients who visited the Drug Rehabilitation Center. After screening, it was determined that the follow-up personnel were suitable for exercise. Data on drug use status and medical health status were extracted in March 2019. Subjects who satisfied the inclusion criteria were assessed for subjective stress, and clinical psychologists judged the patients’ status through structured interviews. In May 2019, a Pre-intervention screening and participation willingness survey was carried out on selected subjects to ensure the application of appropriate exercises and to confirm voluntary participation. A 12-week exercise intervention was performed. Preliminary blood collection, subjective stress assessment, craving questionnaire, sleep quality assessment, and exercise intervention were all conducted in June 2019. Blood collection was carried out immediately after that. A post-test-related content work was carried out in September 2019.

### Physical activity readiness questionnaire+

Using PAR-Q + as a pre-test risk screening tool provides a safe preliminary screening for candidates for exercise testing and exercise prescription. Drug users have a worse physique than the general population. PAR-Q + is a 4-page document containing a series of questions to identify any possible restrictions or limiting participation in physical activities. PAR-Q + is a simple and effective screening tool for physicians, exercise professionals, or participants. First, the participants simply answered seven questions on PAR-Q+page 1. If the answers to all questions were “no”, participants can participate without restriction, following the general physical activity guidelines of a healthy asymptomatic population (35). If the participant answers “yes” to one or more questions, then he or she must complete pages 2 and 3 of the PAR-Q+. Pages 2 and 3 comprise a series of follow-up questions about specific chronic conditions. Page 4 was not answered if the participant was not positive regarding the questions on PAR-Q+pages 2 and 3 about his or her health status and whether the subject may perform physical activities.

### Measurement of craving

#### Perceived stress scale

The Perceived Stress Scale (PSS) is a self-assessed stress scale employed to evaluate individuals’ perceived life stress and uncontrollable experiences within the past month. The questionnaire was scored using a five-point Likert scale that ranges from 0 (“never”) to 4 (“very frequently”). The original scale reverse-scores items in an attempt to represent a total score for perceived stress. Possible scores range from 0 to 40 (0–13 low stress, 14–26 moderate stress, and 27–40 high stress), with higher scores indicating greater stress (α = 0.86) (36).

### Desires for drug questionnaire

The desires for drug questionnaire (DDQ) was initially used to self-evaluate instant cravings for heroin drug abstinent. The DDQ evaluates the craving level in the past month and has the same features as PSS for evaluating subjective stress in the past month. The questionnaire comprises three primary dimensions: desire and intention (the average of the sum of questions 1, 2, 4, 6, 9, 12, and 13; desire and intention: desire for opioids); negative reinforcement (the average of the sum of questions 5, 8, 10, and 11; negative reinforcement: repeated use of heroin to relieve withdrawal symptoms), and control (the average of the sum of questions 3 and 7, control: the ability to control opioids).

### Anxiety self-rating scale

The self-rating scale (SAS) was employed to evaluate the anxiety level of heroin addicts, with 20 items, 15 positive items, and 5 reverse items. 1–4 grades were used, and the results were added to obtain a rough score. After the rough score × 1.25, an integer was used to yield a standard score. SAS > 50 points reflect anxiety.

### Pittsburgh sleep quality index scale

Subjects filled out this scale and assessed their sleep quality through subjective scores. The sleep scale refers to the Pittsburgh Sleep Quality Index Scale (37) compiled by Buysse et al. 37, which is used to assess the subjective sleep quality of the subjects during the past month. A degree test is considered suitable for domestic patients. The PSQI scale is divided into seven factors, each of which is divided into four grades, with the lowest score being 0 and the highest being 3. The higher the total score, the worse the sleep quality.

### Enzyme-linked immunosorbent assay

Blood samples were collected from 7:00 to 8:00 a.m. the day before and the day after the end of the exercise intervention, and an empty stomach was maintained from 8:00 p.m. the day before the blood sampling procedure. Blood samples were collected for OT, AVP, Cortisol, and CRF. A total of 2 ml of venous blood was collected in ADTA anticoagulant tubes, mixed with anticoagulant, and centrifuged at 4,000 rpm for 4 min. The supernatant was stored in an ultra-low temperature freezer at –80°C for further analysis. Blood indicators were detected by the Shanghai Enzyme Link Company.

### Statistical analysis

Statistical analysis of demographic and drug use data was performed using mean ± standard deviation (M ± SD). Data at weeks 0, 6, and 12 were analyzed using a mixed-effects ANOVA test. Group, time, and group*time were analyzed independently. This involved the effect of interaction on subjective stress, craving level, anxiety, sleep quality, and plasma concentrations (OT, AVP, Cortisol, and CRF). The Bonferroni method was applied in the post-test for cases where *p* < *0.05* for pairwise comparisons between groups. Pearson correlation analysis was employed to examine the correlation between subjective stress, craving, negative reinforcement, sleep quality, and anxiety plasma concentrations (OT, AVP, Cortisol, CRF). Statistical analysis was performed using the SPSS 20.0 software, and graphical presentations were illustrated using GraphPad Prism 5 (GraphPad Software, San Diego, CA, USA).

## Results

### Results of perceived stress scale, desires for drug questionnaire, self-rating scale, Pittsburgh sleep quality index scale pre-intervention and post-intervention

**FIGURE 3:**
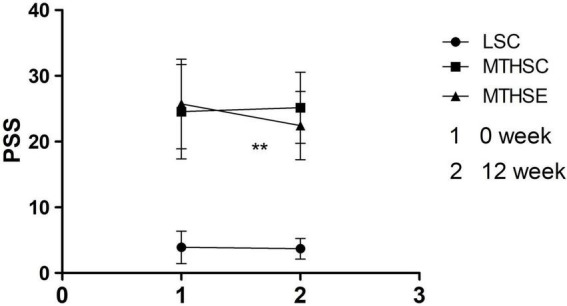
Comparison of perceived stress scale (PSS) scores pre-exercise and post-exercise. 1, Week 0 is the score of PSS; 2, Week 12 is the score of PSS; PSS, self-assessed stress scale; **p < *0.01*, compared with Pre-intervention.

A mixed-effects ANOVA test was used to analyze group and time effects on PSS before and after exercise intervention. The results show that time (*p* < *0.05*, F = 4.750, df = 1) and group (*p* < *0.001*, F = 179.535, df = 2) can significantly affect the degree of subjective stress, and there is a significant interaction between group and time effect (*p* < *0.01*, F = 7.150, df = 2). Before the exercise intervention, the subjective stress level of the LSC group was significantly lower than that of the MTHSC group (95% CI: –24.43, –16.85) and the MTHSE group (95% CI: –25.61, –18.03) (*p* < *0.001*). No difference was reported between MTHSC and MTHSE groups (*p* > 0.05, 95% CI: –2.64, –5.00). After the exercise intervention, the subjective stress level of the MTHSE group was significantly lower than before the exercise (*p* < *0.001*, 95% CI: 1.756, 4.815) (Figure 3).

**FIGURE 4:**
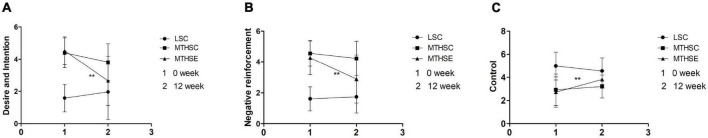
Comparison of desires for drug questionnaire (DDQ) scores pre-exercise and post-exercise. 1, Week 0 is the score of “Desire and Intention”, “Negative reinforcement” and “Control”; 2, Week 12 is the score of “Desire and Intention”, “Negative reinforcement” and “Control”; Desire and Intention, desire for heroin; Negative reinforcement, repeated use of heroin to relieve withdrawal symptoms; Control, the ability to control heroin; **p < *0.01*, compared with Pre-intervention.

A mixed-effects ANOVA test was used to analyze group and time effects on DDQ before and after exercise intervention. The results show that time can significantly affect “Desire and Intention” (*p* < *0.001*, F = 27.38, df = 1) and “Negative reinforcement” (*p* < *0.001*, F = 22.38, df = 1), as well as group “Desire and Intention” (*p* < *0.001*, F = 179.535, df = 2) and “Negative reinforcement” (*p* < *0.001*, F = 179.535, df = 2) and “Control” (*p* < *0.001*, F = 35.141, df = 2). A significant interaction effect exists between group and time (*p* < *0.001*, F = 19.18, df = 2; *p* < *0.01*, F = 8.16, df = 2; *p* < *0.01*, F = 6.73, df = 2). Before the exercise intervention, the “Desire and Intention” and “Negative reinforcement” of LSC group were significantly lower than those of MTHSC group (95% CI: −3.51, –2.37; 95% CI: –3.54, –2.36) and MTHSE group (95% CI: –25.61, –18.03; 95% CI: –3.25, –2.07) (*p* < *0.01*). “Control” was significantly higher than MTHSC group (95% CI: 1.22, 2.89) and MTHSE group (95% CI: 1.42, 3.09) (*p* < *0.01*). No difference was reported between the MTHSC group and the MTHSE group between “Desire and Intention” and “Negative reinforcement”, “Control” (*p* > 0.05). After the exercise intervention, the subjective of the “Desire and Intention” (*p* < *0.001*, 95% CI: 1.60, 2.71) and “Negative reinforcement” (*p* < *0.001*, 95% CI: 0.85, 1.90) in MTHSE group were significantly lower than before exercise, and “Control” was significantly higher than before exercise (*p* < *0.001*, 95% CI: –1.68, –0.50). The “Desire and Intention” (95% CI: 0.57, 2.26) and “Negative reinforcement” (95% CI: 0.52, 2.15) of the MTHSE group were significantly lower than those of the MTHSC group (Figure 4).

**FIGURE 5:**
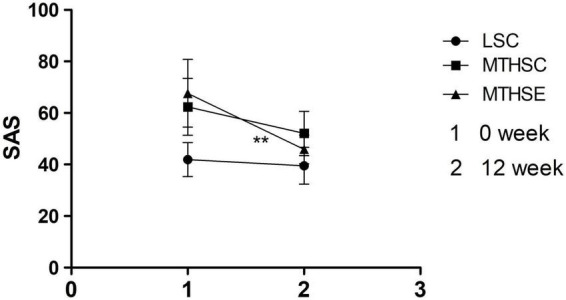
Comparison of self-rating scale (SAS) scores pre-exercise and post-exercise. 1, Week 0 is the score of SAS; 2, Week 12 is the score of SAS; SAS, Anxiety level; **p < *0.01*, compared with Pre-intervention.

A mixed-effects ANOVA test was used to analyze group and time effects on SAS before and after exercise intervention. The results show that both time (*p* < *0.001*, F = 86.65, df = 1) and group (*p* < *0.001*, F = 50.93, df = 2) can significantly affect anxiety levels. A significant interaction was reported between group and time effect (*p* < *0.001*, F = 20.99, df = 2). Before exercise intervention, the anxiety level of LSC group was significantly lower than that of MTHSC group (95% CI: –27.34, –13.66) and MTHSE group (95% CI: –32.59, –18.91) (*p* < *0.001*). No difference was reported between MTHSC and MTHSE groups (*p* > 0.05, 95% CI: –12.15, 1.65). After exercise intervention, MTHSC group (*p* < *0.001*, 95%CI: 6.05, 14.60) and MTHSE group (*p* < *0.001*, 95% CI: 17.51, 26.06) had significantly lower anxiety levels than before exercise, while MTHSE group had significantly lower anxiety levels than the MTHSC group (95% CI: 1.53, 10.91) (Figure 5).

**FIGURE 6:**
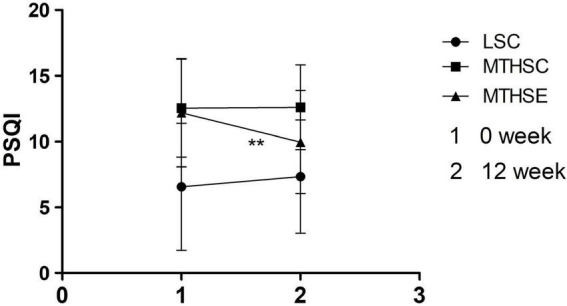
Comparison of Pittsburgh sleep quality index scale (PSQI) scores pre-exercise and post-exercise. 1, Week 0 is the score of PSQI; 2, Week 12 is the score of PSQI; PSQI, sleep quality; **p < *0.01*, compared with Pre-intervention.

A mixed-effects ANOVA test was used to analyze group and time effects on PSQI total score before and after exercise intervention. The results showed that group (*p* < *0.001*, F = 16.635, df = 2) could significantly affect sleep quality, and there was a significant interaction effect between group and time (*p* < *0.001*, F = 9.21, df = 2). For exercise before intervention, the total PSQI score of LSC group was significantly lower than that of the MTHSC group (95% CI: −8.744, −3.23) and MTHSE group (95% CI: –8.38, –2.88) (*p* < *0.001*). No difference was reported between them (*p* > 0.05, 95% CI: –2.42, 3.13). After exercise intervention, the total PSQI score of MTHSE group (*p* < *0.001*, 95% CI: 1.18, 3.25) was significantly lower than before exercise. The total PSQI score of MTHSE group was significantly lower than that of the MTHSC group (*p* < *0.05*, 95% CI: 0.136, 5.15) (Figure 6).

### Pre-intervention and post-intervention plasma oxytocin, vasopressin and cortisol levels

**FIGURE 7:**
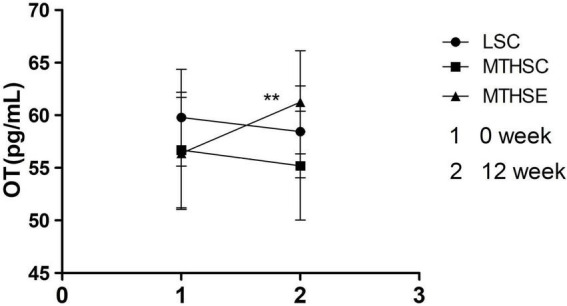
The effect of exercise on plasma oxytocin (OT) concentration. 1, Plasma OT concentration at week 0; 2, Plasma OT concentration at week 12; **p < *0.01*, compared with Pre-intervention.

A mixed-effects ANOVA test was used to analyze group and time effects on OT before and after exercise intervention. The results showed that group (*p* < *0.01*, F = 6.67, df = 2) could significantly affect plasma OT levels, and there was a significant interaction effect between group and time (*p* < *0.01*, F = 7.72, df = 2). Before exercise intervention, there was no difference between MTHSC and MTHSE groups (*p* > 0.05, 95% CI: –3.03, 3.681). After exercise intervention, the plasma OT level of MTHSE group (*p* < *0.001*, 95% CI: –7.48, –2.26) was significantly higher than that before exercise, and the plasma OT level of MTHSE group was significantly higher than that of the MTHSC group (95% CI: –9.18, –2.90) (Figure 7).

**FIGURE 8:**
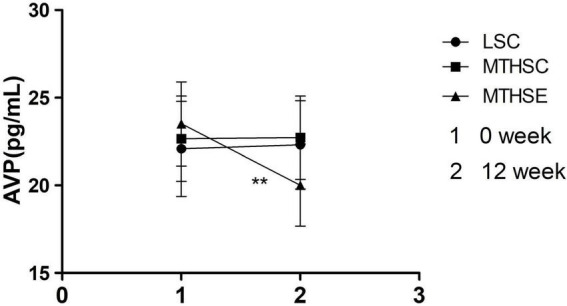
The effect of exercise on plasma vasopressin (AVP) concentration. 1, Plasma AVP concentration at week 0; 2, Plasma AVP concentration at week 12; **p < *0.01*, compared with Pre-intervention.

A mixed effects analysis of variance was employed to investigate the effects of group and time on plasma AVP levels before and after exercise intervention. The results showed that time (*p* < *0.001*, F = 37.79, df = 1) could significantly affect plasma AVP levels. A significant interaction effect occurred between group and time (*p* < *0.001*, F = 48.6, df = 2). In terms of exercise before the intervention, no difference was reported between the MTHSC group and the MTHSE group (*p* > 0.05, 95% CI: –2.484, 0.796). After the exercise intervention, the plasma AVP of the MTHSE group (*p* < *0.001*, 95% CI: 2.90, 4.10) was significantly lower that before exercise. And the plasma AVP level of MTHSE group was significantly lower than that in the MTHSC group (*p* < *0.001*, 95% CI: 1.14, 4.29) and the LSC group (*p* < *0.01*, 95% CI: 0.74, 3.86) (Figure 8).

**FIGURE 9:**
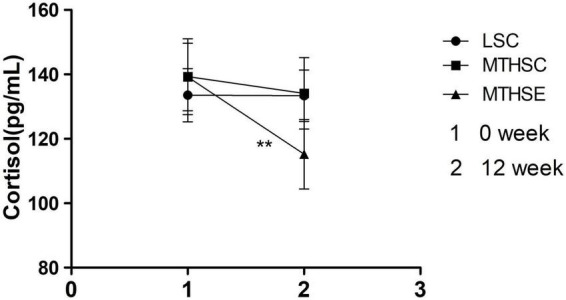
The effect of exercise on plasma cortisol concentration. 1, Plasma Cortisol concentration at week 0; 2, Plasma Cortisol concentration at week 12; **p < *0.01*, compared with Pre-intervention.

A mixed effects analysis of variance was used to investigate the effects of group and time on plasma Cortisol levels before and after exercise intervention. The results showed that both time (*p* < *0.001*, F = 64.93, df = 1) and group (*p* < *0.001*, F = 9.15, df = 2) could significantly affect plasma cortisol levels. A significant difference took place between group and time. Before exercise intervention, no difference was reported between MTHSC group MTHSE groups (*p* > 0.05, 95% CI: –6.56, 6.79). After exercise intervention, the MTHSC group (*p* < *0.05*, 95% CI: 0.99, 9.40) and MTHSE group (*p* < *0.001*, 95% CI: 19.76, 28.17) had significantly lower plasma cortisol levels, and MTHSE group had significantly lower plasma Cortisol levels than the MTHSC group (*p* < 0.001, 95% CI: 12.36, 25.41) and the LSC group (*p* < *0.01*, 95% CI: 11.69, 24.63) (Figure 9).

### The results of correlation analysis between psychological scale and blood index

Pearson correlation analysis was employed to explore the correlation between various psychological indicators and blood indicators. The results showed that OT was negatively correlated with PSS (*r* = –0.491, *p* < *0.001*) and cortisol (*r* = −0.452, *p* < *0.001*), respectively. A positive correlation was observed with PSS (*r* = 0.525, *p* < *0.001*). “Desire and Intention” (*r* = 0.682, *p* < *0.001*), “negative reinforcement” (*r* = 0.673, *p* < *0.001*), SAS (*r* = 0.649, *p* < 0.001), PSQI (*r* = 0.431, *p* < *0.001*) were positively correlated with PSS, respectively. OT was associated with “Desire and Intention” (*r* = –0.491, *p* < *0.001*), “Negative reinforcement” (*r* = –0.363, *p* < 0.05), SAS (*r* = –0.232, *p* < *0.05*), and AVP (*r* = –0.317, *p* < *0.01*) (Table 2).

**TABLE 2:** Correlation analysis results of various dimensions between psychological scale and blood index.

	PSS	Desire and Intention	Negative reinforcement	SAS	PSQI	OT	AVP	Cortisol
PSS	*r* = 1	*r* = 0.682** *p* < *0.001*	*r* = 0.67** *p* < *0.001*	*r* = 0.649** *p* < *0.001*	*r* = 0.431** *p* < *0.001*	*r* = −0.491** *p* < *0.001*	*r* = 0.145 *p* = 0.184	*r* = 0.525** *p* < *0.001*
Desire and Intention		*r* = 1	*r* = 0.756** *p* < *0.001*	*r* = 0.595** *p* < *0.001*	*r* = 0.474** *p* < *0.001*	*r* = −0.363** *p* < *0.001*	*r* = 0.237* *p* < *0.001*	*r* = 0.221* *p* < *0.05*
Negative reinforcement			*r* = 1	*r* = 0.564** *p* < *0.001*	*r* = 0.476** *p* < *0.001*	*r* = −0.228** *p* < *0.05*	*r* = 0.098 *P* = 0.373	*r* = 0.26* *p* < *0.05*
SAS				*r* = 1	*r* = 0.421** *p* < *0.001*	*r* = −0.232* *p* < *0.05*	*r* = 0.174 *p* = 0.212	*r* = 0.247* *p* < *0.05*
PSQI					*r* = 1	*r* = −0.152 *p* = 0.164	*r* = 0.072 *p* = 0.51	*r* = 0.234* *p* < *0.05*
OT						*r* = 1	*r* = −0.317* *p* < *0.01*	*r* = −0.452** *p* < *0.001*
AVP							*r* = 1	*r* = 0.029 *p* = 0.79
Cortisol								*r* = 1

**Medium high significance correlation; *Low significant correlation.

## Discussion

This prospective study explored the peripheral biomarkers of stress in opioid addicts based on the HPA axis to investigate the relationship between peripheral plasma oxytocin, vasopressin, cortisol, etc. Our findings support our hypothesis based on the correlations between craving levels, assuming that exercise can modulate changes in peripheral biomarkers and correspondingly improve self-perceived stress and craving levels.

The odds of relapse due to stress are high in people with opioid use disorder, and stress is believed to have an important role in the development, maintenance, and relapse of addiction by increasing cravings and motivation (38). The relationship between stress and addiction may be bidirectional, as individuals with pre-existing drug addiction are highly likely to choose to use drugs to escape stress, and chronic stress is a key factor in addiction (39). From the perspective of chronic stress, the selected psychological questionnaire is also a measure of subjective perceived stress, craving, and negative reinforcement within the past month. Through Pearson correlation analysis, the subjective perception stress was positively correlated with the degree of craving (*r* = 0.682, *p* < *0.001*) and negative reinforcement (*r* = 0.673, *p* < *0.001*), while the degree of craving related to negative reinforcement (*r* = 0.756, *p* < *0.001*) was positively correlated. The correlation coefficients were high, and the consistency between the dimensions was good. This also proves that opioid addicts’ drug cravings are closely related to their emotional states. Interestingly, through the survey data display and correlation analysis, we found that the lower stress subjects have the same lower level of craving, negative reinforcement and anxiety, while the higher stress subjects have the same higher level of craving, negative reinforcement and anxiety. This may be related to the sensitivity of individual self-regulation in the environment of the rehabilitation center. The subjects with lower stress can better self-regulation, so that they have less stress, less negative reinforcement and less craving than others in the baseline state, and can maintain a good state with the passage of time in the rehabilitation center.

In recent years, peripheral oxytocin administration has emerged as a potential treatment for addiction. OT can suppress opioid tolerance (40), reduce opioid self-administration, and reduce cravings and stress responses in opioid addicts (41). These studies are based on the association of peripheral oxytocin with many symptoms of drug addicts. A survey study found a negative correlation between plasma OT levels and negative reinforcement and stress in selecting relevant indicators for drug addicts (42). Heroin addicts have negative emotional experiences during withdrawal, characterized by restlessness, irritability, anxiety, and abnormal stress responses that induce drug-seeking behavior. These addicts exhibit elevated stress responses during withdrawal and are associated with drug cravings (43). We studied the relationship between peripheral plasma OT and addiction related behaviors in opioid addicts, and found that plasma OT was negatively correlated with stress and desire, respectively. Negative reinforcement and anxiety were also negatively correlated. Although peripheral OT measures are only a marker of what might be going on at the level of the hyperpatharamus, which might not even be the brain region where these stress related responses are being mediated, and this data is also relevant in nature, and there are also changes in receiver expression in targeted addiction, it seems OXTr may be over-expressed, and expression of peptide and receptor is dynamically regulated.

The positive effects of OT on stress-related hormones are particularly important in the treatment of addiction. Chronic drug abuse causes behavioral changes associated with neuroplasticity and neurochemical changes in stress-related brain regions (44, 45). Since we cannot obtain OT values for the central nervous system of the brain, peripheral OT was performed based on associations with stress- and addiction-related symptoms. While there has been debate as to whether central and peripheral OT are related (45, 46), animal research has revealed coupled central and peripheral OT release during stress (47) and axonal projections from magnocellular OT neurons to forebrain structures, the amygdala, and the posterior pituitary (48). These studies have also reported an increase in OT release to the peripheral and cerebral central nervous system, accompanied by a decrease in fear-related behaviors mediated by the amygdala (48). In humans, peripheral OT has been shown to rise during stress, possibly promoting relationship repair in response to interpersonal stress (49, 50). The function of OT in the treatment of drug addiction is also obvious. Our research group also found in previous studies that the up-regulation of peripheral plasma oxytocin can potentially improve social anxiety and cue-induced cravings in female heroin addicts to a certain extent (32). Clinical and pre-clinical evidence shows that OT can also improve symptoms associated with depression, such as sleep disturbance (51). Our study did not find the same results by correlating OT with PSQI total scores. The above shows that OT may not be highly correlated with sleep quality in opioid addicts.

Stress and anxiety-inducing stimuli activate the central and peripheral release of OT, which is associated with the inhibition of the basal and stress-induced hormonal activity of the hypothalamic-pituitary-adrenal (HPA) axis (52) and the endogenous OT system in stressful situations. The activation of OT has led researchers to consider the extent to which OT administration can reduce stress and anxiety in animals and humans (52, 53). The stress-regulating effects of chronic OT work by reducing stress and HPA axis-related hormones in animals [e.g., cortical ketone, adrenocorticotropic hormone (ACTH)] was further demonstrated (19, 20, 54–56). We also found that the peripheral plasma OT of opioid addicts was negatively correlated with cortisol (*r* = –0.452, *p* < *0.001*) and subjectively perceived stress (*r* = –0.491, *p* < *0.001*). The two coefficients were relatively high and relatively close. Simultaneously, detecting plasma cortisol as stress also provides us with guidance. There was a positive correlation between the two (*r* = –0.525, *p* < *0.001*), and the correlation coefficient was high. This close association provides the basis for our prediction of oxytocin and cortisol as biomarkers of peripheral stress in opioid addicts and for improving subjectively perceived stress by modulating peripheral oxytocin or cortisol. Peripheral OT likely acts on the HPA axis to alter cortisol, leading to positive changes in subjective stress levels, possibly because OT-related changes in receptor expression, binding, and peripheral hormone levels can inhibit HPA axis activity (19, 20, 54, 55, 57, 58) and prevent its up-regulation in stressful situations (19, 59). At the same time, the changes of OT, AVP and cortisol in peripheral plasma caused by subjective pressure have also been confirmed in scholars’ studies that pressure will activate the sympathetic nervous system, which releases catecholamine and starts a coordinated biochemical reaction, leading to the increase of oxytocin, vasopressin, and corticotropin releasing factor (60, 61). At the same time, pressure also up-regulates the hypothalamus pituitary adrenal (HPA) axis and inflammatory activities, such as the change of cortisol (62). This also verifies the high correlation between subjective pressure and plasma OT, AVP, cortisol before exercise.

Oxytocin, and vasopressin are widely applied in research to investigate drug addiction as two interacting substances. The role of OT has been broadly studied, and AVP is also a part of the focus of this study. Opioids and their analogs not only interact with anterior pituitary hormones but also affect the posterior pituitary (neurohypophysis). AVP and OT are individually and synergistically affected by opioids. Numerous studies use plasma OT and AVP as mood variables to reflect central levels, and lower plasma OT and higher AVP levels are associated with various psychiatric disorders (63–65). Correlation analysis reported that plasma OT was significantly associated with AVP, with a negative correlation (*r* = –0.317, *p* < *0.01*). The long-term administration of opioids such as morphine has shown changes in OT expression in various regions in the brain, including a decrease in OT in SON and NAc, as well as an increase in VTA (66). Increased AVP gene expression in the amygdala is associated with early withdrawal from chronic opioid exposure (67). Through the correlation analysis between plasma AVP and craving, we found that the two were positively correlated (*r* = 0.363, *p* < 0.001), which was similar to the results of those in previous studies. Simultaneously, prior studies have shown that after taking opioids, the AVP of the hypothalamus that activates the V1b receptor stimulates the secretion of ACTH by adrenocorticotropic hormone in the anterior pituitary (68, 69) in response to stress. Through analysis, we found no correlation between plasma AVP and subjectively perceived stress, and we did not select ACTH as an indicator to investigate the peripheral mechanism of plasma AVP acting on stress, which is also a limitation of this study.

The results obtained in this study showed that 12 weeks of aerobic combined with resistance exercise increased plasma OT levels, decreased plasma AVP and cortisol levels, reduced subjectively perceived stress and craving levels, and improved sleep quality in male opioid addicts. Moreover, the improvement effect of exercise has been proven. Exercise can improve the level of stress and regulate the function of various biochemical indicators of the HPA axis. Animal studies on drug addiction have shown that exercise-boosted testosterone secretion suppresses the HPA axis in male rats (69, 70), but this has not been demonstrated in human studies. We speculate that the increase of testosterone in male opioid addicts after exercise may be effective in suppressing the HPA axis, but we did not obtain testosterone indicators, which is also a limitation of this study and will be supplemented in future work. Other studies have also recognized that exercise modulates biomarkers such as peripheral OT and improve cravings in drug addicts (32). This study was primarily based on the HPA axis, which may be because exercise can regulate OT levels in blood and hypothalamus, while changes in blood OT and OT levels in the hypothalamic and extra-hypothalamic brain regions can increase OT. mRNA levels (71–73). OT helps to normalize the HPA axis and reduce stress-related physiologic biochemistry (e.g., cortisol), subjective responses (e.g., anxiety, craving, and sleep quality), etc. As a biochemical substance with opposite effect to OT, AVP also has significant effect. At the same time, exercise can improve the pressure and mood of drug addicts by corresponding AVP level. Of course, this is based on a study by Vendruscolo et al. (74) which found that blocking glucocorticoid itself may be a potential treatment addiction, while exercise may regulate AVP level to a certain extent to block glucocorticoid and reduce drug seeking of drug addicts (75).

In addition to the shortcomings mentioned above, this study still has some shortcomings. For example, only males were selected, but female dependents were not selected. To some extent, gender differences have not been taken into account and will be compared in future studies. At the same time, this study only measured biomarkers, and it would be more convincing if the analysis of the structure of relevant brain regions were added. In future research, we will explore the improvement of stress in drug addicts based on a variety of exercise intervention modalities and personalized exercise intensity prescriptions. Also enriched with measures of cortisol indicators, such as urine, saliva, hair, etc., to enhance the illustration of biomarkers of stress. Based on the results of this study, a certain hypothesis was put forward, that is, the brain pressure brain area and peripheral hormones will have the same changes, which may be because exercise regulates the hormones related to pressure, first changes the brain central nervous system, and then leads to changes in the hormones related to peripheral pressure and addiction, and then reduces the pressure and craving of addicts. The FMRI test was added to measure the structural changes of relevant brain regions, and the correlation between them and biomarkers was constructed to jointly explain the problem.

## Conclusion

As an auxiliary treatment, exercise improves the plasma OT, AVP and cortisol levels of opioid addicts, and reduces their subjective perceived stress level, desire, negative reinforcement level, anxiety level, and sleep quality. In addition, peripheral plasma OT, AVP, and cortisol may play a role as potential peripheral biomarkers to predict stress in male opioid addicts.

## Data availability statement

The original contributions presented in this study are included in this article/supplementary material, further inquiries can be directed to the corresponding author.

## Ethics statement

This study complied with the Declaration of Helsinki and was approved by the Ethics Committee of Guangxi Minzu University (Batch number 16-2010). The patients/participants provided their written informed consent to participate in this study.

## Author contributions

JW screened experimental subjects, signed the informed consent process, and conducted the exercise intervention. Both authors conceived and designed the experiments, contributed to the article, and approved the submitted version.
